# Tapetal 3-Ketoacyl-Coenzyme A Synthases Are Involved in Pollen Coat Lipid Accumulation for Pollen-Stigma Interaction in *Arabidopsis*

**DOI:** 10.3389/fpls.2021.770311

**Published:** 2021-11-23

**Authors:** Zaibao Zhang, Huadong Zhan, Jieyang Lu, Shuangxi Xiong, Naiying Yang, Hongyu Yuan, Zhong-Nan Yang

**Affiliations:** ^1^College of Life Science, Xinyang Normal University, Xinyang, China; ^2^Shanghai Key Laboratory of Plant Molecular Sciences, College of Life Sciences, Shanghai Normal University, Shanghai, China; ^3^State Key Laboratory of Crop Genetics and Germplasm Enhancement, College of Agriculture, Nanjing Agricultural University, Nanjing, China

**Keywords:** pollen coat, lipid, 3-ketoacyl-coenzyme A synthase, pollen hydration, anther, very long chain fatty acid

## Abstract

Pollen coat lipids form an outer barrier to protect pollen itself and play essential roles in pollen-stigma interaction. However, the precise molecular mechanisms underlying the production, deposition, regulation, and function of pollen coat lipids during anther development remain largely elusive. In lipid metabolism, 3-ketoacyl-coenzyme A synthases (KCS) are involved in fatty acid elongation or very-long-chain fatty acid (VLCFA) synthesis. In this study, we identified six members of the *Arabidopsis* KCS family expressed in anther. Among them, KCS7, KCS15, and KCS21 were expressed in tapetal cells at anther stages 8–10. Further analysis demonstrated that they act downstream of male sterility 1 (MS1), a regulator of late *tapetum* development. The *kcs7/15/21* triple mutant is fertile. Both cellular observation and lipid staining showed pollen coat lipid was decreased in *kcs7/15/21* triple mutant. After landing on stigma, the wild-type pollen grains were hydrated for about 5 min while the *kcs7/15/21* triple mutant pollen took about 10 min to hydrate. Pollen tube growth of the triple mutant was also delayed. These results demonstrate that the *tapetum*-localized KCS proteins are involved in the accumulation of pollen coat lipid and reveal the roles of tapetal-derived pollen coat lipid for pollen-stigma interaction.

## Introduction

Pollen-pistil interaction is critical for the successful fertilization of flowering plants. This interaction is a crucial step in preventing inbreeding and maintaining species identity, thus contributing to angiosperm diversity. It consists of multiple selective steps, including pollen adhesion, hydration, germination, and polarized tube growth (Edlund et al., 2004; Bedinger et al., 2017; Zheng et al., 2018). Pollen adhesion and hydration are the two earliest events in pollination among species with dry stigmas. These highly regulated processes require proteins and lipids deriving from the pollen wall (Elleman and Dickinson, 1990; Safavian and Goring, 2013).

The mature pollen wall of flowering plants includes three main layers, namely intine, exine, and pollen coat. The development of the pollen wall is controlled by transcription factors, namely, dysfunctional tapetum 1 (DYT1), defective in tapetal development and function 1 (TDF1), aborted microspores (AMS), MS188, and male sterility 1 (MS1), which form a genetically defined regulatory pathway: *DYT1-TDF1-AMS-MS188-MS1* (Zhu et al., 2011). Loss of these transcription factors results in male sterility. Intine is produced by microspores and is mainly composed of pectin, cellulose, and hemicellulose. Exine is composed of sexine and nexine (Lou et al., 2014). The main composition of sexine is sporopollenin. Its precursors are synthesized by multiple enzymes regulated by the v-myb avian myeloblastosis viral oncogene homolog (MYB) transcription factor MS188 in *tapetum* (Zhang et al., 2007; Wang et al., 2018). A pollen coat contains many sticky and heterogeneous materials composed of lipids, proteins, carotenoids, and polysaccharides (Piffanelli et al., 1998; Hernández-Pinzón et al., 1999), which is crucial for the pollen protection from abiotic stresses, successful pollen contact, hydration, and subsequent pollen germination among species with dry stigmas (Elleman and Dickinson, 1990; Dickinson, 1995). It also contains various components, including pollen coat proteins (P) and pollen coat lipids. PCPs are derived from *tapetum* after it undergoes programmed cell death (PCD) alongside gametophytically derived proteins. In *Arabidopsis*, several lipases and lipid-binding oleosin proteins were identified as PCPs (Mayfield et al., 2001). MS1 is a plant homeodomain motif (PHD) transcription factor for late *tapetum* development (Wilson et al., 2001). The sporophyte-derived PCPs are regulated by MS1 in *tapetum* (Lu et al., 2020), and these PCPs may play essential roles in the early events of pollen-stigma recognition including pollen adhesion and hydration. S-locus cysteine-rich (SCR) protein, SLR1-BP1/2, PCP-A1 (PCP7), and PCP-Bs have been identified as gametophytically derived PCPs (Doughty et al., 1998; Takayama et al., 2000; Nasrallah and Nasrallah, 2014; Wang et al., 2017). They are likely to play important roles in pollen germination and pollen tube growth.

Lipids are one of the major subcellular components and comprise different combinations and positional distributions of fatty acids. They play important roles in plants by forming membrane structures, acting as storage lipids, signaling molecules, and covering the surface (Li-Beisson et al., 2016). The pollen coat lipids display a semisolid state and may regulate the water transfer from the stigma to pollen during the pollen-stigma interaction (Edlund et al., 2004). In lipid metabolism, 3-ketoacyl-coenzyme A (CoA) synthases (KCS) are involved in fatty acid elongation or very-long-chain fatty acid (VLCFA) synthesis (Joubès et al., 2008; Haslam and Kunst, 2013). KCS catalyzes the condensation of the C2 carbon moiety to acyl CoA in the elongation of VLCFA-CoAs, which are further catalyzed by fatty acid hydroxylases (CER1, CER3) to form precursors of wax and cutin (Li-Beisson et al., 2016). Eceriferum 2 (CER2) and CER2-like 2 (CER2l2) encode BEAT, benzylalcohol O-acetyltransferase; AHCT, anthocyanin O-hydroxycinnamoyltransferase; HCBT, anthranilate N-dydroxycinnamoyl; DAT, deacetylvindoline 4-O-acetyltransferase (BAHD) acyltransferase and enhance the elongation of VLCFA-CoAs from 28 to 30 carbon atoms catalyzed by KCS6 (Fiebig et al., 2000; Haslam et al., 2015). The *kcs6* single mutant and *cer2cer2l2* double mutant can produce mature pollen. However, they fail to hydrate on stigma (Preuss et al., 1993; Hulskamp et al., 1995; Fiebig et al., 2000; Haslam et al., 2015). KCS6, CER2, and CER2L2 were expressed in endothecium suggesting that endothecium plays a role in synthesizing pollen coat lipids for pollen hydration (Zhan et al., 2018). It was generally believed that pollen coat lipids are derived from *tapetum* (Hernández-Pinzón et al., 1999). It is not clear how *tapetum* contributes to the synthesis of pollen coat lipids.

The KCS family in *Arabidopsis* contains 21 *KCS* members (Costaglioli et al., 2005; Joubès et al., 2008). FAE1/KCS18 is the first identified KCS, which is specifically expressed in seed, and involved in the synthesis of the seed storage lipids (James et al., 1995; Millar and Kunst, 1997). *KCS2/DAISY*, *KCS20*, *KCS9*, and *KCS10/FDH1* are shown to be involved in the synthesis of VLCFAs for cuticular waxes (Lolle et al., 1997; Yephremov et al., 1999; Franke et al., 2009; Kim et al., 2013). KCS6/CER6/CUT1 is involved in the elongation of fatty acyl-CoAs longer than C28 VLCFA for cuticular waxes in both epidermis and pollen coat (Millar and Kunst, 1999; Fiebig et al., 2000; Hooker et al., 2002). Mutation in *KCS6* leads to humidity-sensitive genic male sterility (HGMS) by affecting pollen coat lipids (Zhan et al., 2018). However, the functions of other *KCS* genes are not clearly understood. In this study, we reported *KCS7*, *KCS15*, and *KCS21* were expressed in the *tapetum*. They act downstream of MS1, a regulator for late *tapetum* development. The reduced pollen coat lipid in the triple mutant (*kcs7/15/21*) and defective pollen hydration demonstrate that they play redundant roles in pollen coat lipid synthesis for pollen hydration.

## Materials and Methods

### Plant Materials and Growth Conditions

*Arabidopsis thaliana* plants were grown in a greenhouse at 21°C and 16-h light/8-h dark photoperiod. T-DNA insertion mutant of *kcs7* (*At1g71160*: *kcs7-1*, SAIL_207_H11; *kcs7-2*, SALK_023400), *kcs15* (*At3g52160*, SALK_209946C), and *kcs21* (*At5g49070*: *kcs21-1*, SALK_005116; *kcs21-2*, SALK_089611) in Col-0 background were all obtained from the ABRC stocks. T-DNA insertion status of *KCS7*, *KCS15*, and *KCS21* was confirmed using the T-DNA border primer in combination with gene-specific primers listed in Supplementary Table 1.

### Phylogenetic Analyses

HMMER search algorithm (*E*-value = 1e−10) and NCBI Basic Local Alignment Search Tool algorithm (BLASTP) (*E*-value = 1e−10) were used to search for KCS proteins (Finn et al., 2016). The protein sequences from *A. thaliana*, *Capsella grandiflora*, *Carica papaya*, *Oryza sativa*, *Zea mays*, *Amborella trichopoda*, *Ginkgo biloba*, *Physcomitrella patens*, and *Chlamydomonas reinhardtii* genomes were extracted from Phytozome v10^1^ and were further verified using the SMART database.^2^ The full-length amino acids were used to construct a phylogenetic tree with the maximum likelihood (ML) method based on the JTT amino acid substitution model.

### RNA Extraction and Quantitative Reverse Transcription-PCR

The total RNA was isolated using TRIzol reagent (Invitrogen, Thermo Fisher Scientific, United States) according to the instructions of the manufacturer. The RNA quantity and purity were assessed using NanoQuant Spectrophotometer (Tecan, Switzerland). The first-strand cDNAs were synthesized from DNase I-treated total RNA using the cDNA Synthesis Kit (Takara, Japan). The gene expression was normalized using tubulin beta 8 (TUB8) (At5g23860) as the reference gene (Gu et al., 2014), and the relative gene expression was calculated as the mean of three biological replicates and three technical replicates. Gene-specific primers used for quantitative reverse transcription-PCR (qRT-PCR) are listed in Supplementary Table 1.

### Microscopic and Phenotypic Characterization

Pollen viability was assessed using Alexander’s staining. Stage 12 anthers were collected and stained with Alexander’s solution overnight at room temperature. Then, the anthers were photographed using a microscope when the active pollen grains were stained red. For scanning electron microscopic (SEM) analysis, mature pollen grains from freshly dehisced anthers were mounted and coated with gold on stubs. The samples were then immediately observed using FESEM (SU8010, Japan). Transmission electron microscopy (TEM) analysis was performed as described previously (Zhang et al., 2007). Different stage anthers were fixed in 0.1 M phosphate buffer (pH 7.2) with 2.5% glutaraldehyde (v/v) buffer and embedded into resin. Ultrathin sections (70 nm thick) were cut, counterstained with 2% uranyl acetate in 100% methanol and 1% dimethyl sulfoxide, and observed using a TEM microscope (JEOL, Japan). For pollen coat deposition analysis, more than 20 anthers were cut and photographed using TEM. The procedure of semithin sectioning and lipid staining was performed as described previously (Zhang et al., 2007; Lou et al., 2014). Semi-sections (1 μm thick) were stained using Tinapol (Sigma, United States) for 15 min (10 μg/μl) and diethyloxadicarbocyanine iodide (DiOC_2_) for 5 min (5 μg/ml), and then were photographed using Olympus BX51 Microscope (Japan) using 390–440 nm excitation filter and 478 nm blocking filter.

### Protein Localization Analysis

For protein localization analysis, the full genomic sequences including the promoter of *KCS* genes were cloned and ligated with the p1300-GFP vector (Supplementary Table 1). The recombined plasmids were transformed into *col* wild-type (WT) *Arabidopsis* using the floral dip method (Clough and Bent, 1998), and the transgenic plants were screened using 20 mg L^–1^ hygromycin. The flower buds of the T_2_ transgenic plants were harvested, and the different stage anthers were isolated, and the green fluorescent protein (GFP) fluorescence was scanned using Zeiss LSM 510 confocal scanning microscope. The GFP excitation was detected at 488 nm, and the emitted light was detected at 520 nm. Ten anthers were analyzed for each stage.

### Semi-*in vivo* Pollen Hydration and Pollen Tube Growth Assay

For pollen hydration, freshly opened *ms188* pistil was cut and attached upright on agar (1.0%). Then, pollen grains (*n* = 50–80) from a freshly dehiscing anther were transferred onto the stigmatic papilla. The behavior of the pollen was captured under a microscope immediately after pollinations were initiated. For *in vivo* pollen tube growth analysis, pollinations were initiated on *ms188* stigmas and allowed to proceed for 2 h as described previously (Wang et al., 2017). Then, stigmas were fixated overnight (60% v/v ethanol, 30% v/v chloroform, and 10% v/v acetic acid), incubated in 8 M NaOH for 20 min, and washed in dH_2_O three times (5 min/each). Samples were dyed in 0.1% decolorized aniline blue (0.1% w/v aniline blue in 0.1 M K_3_PO_4_, pH11) for 1 h, and images were captured using an Olympus BX51 microscope (Japan). Pollen tube growth was calculated as the mean of three biological replicates. All statistical analyses were carried out using Microsoft Excel.

### Statistical Analysis

More than 500 pollen grains were photographed using SEM, and the aborted pollen grains were numbered. The relative fluorescence fold change of DiOC_2_ was quantified by measuring 80 pollen grains from 6 differential anther sections using Quantity One software (Bio-Rad, United States). The relative fold change of pollen coat concentration was quantified by measuring 60 pollen grains from 20 differential TEM sections using Quantity One software (Bio-Rad, United States). One-way ANOVA or Student’s *t*-test was used to evaluate the statistical significance between different genotypes. Different lowercase letters above the brackets represent statistically significant differences.

## Results

### Six *KCS* Genes Express in Anther of *Arabidopsis*

**FIGURE 1:**
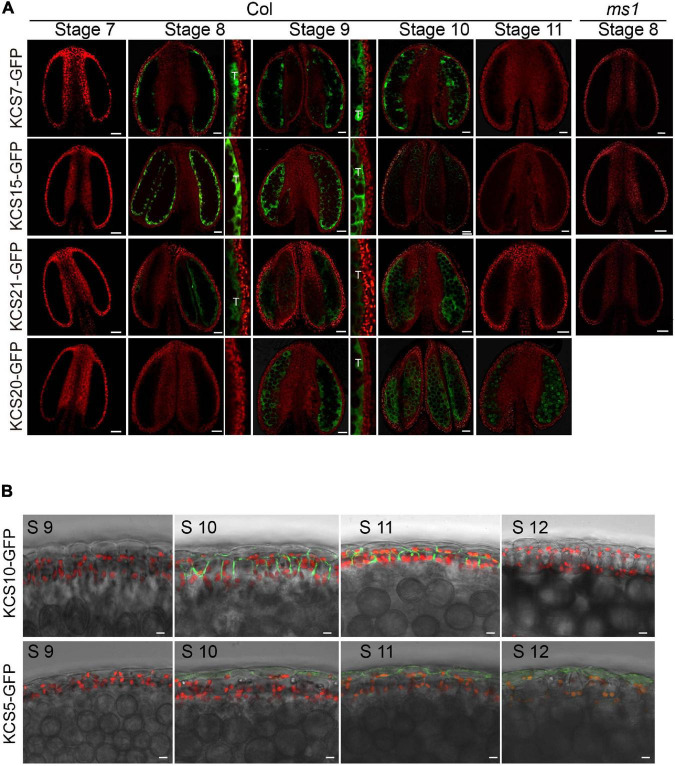
Expression analysis of KCS proteins during anther development. **(A)** The expression of KCS7-GFP, KCS15-GFP, and KCS21-GFP fusion proteins were initially detected in *tapetum* at stage 8 and subsequently accumulated in *tapetum* and anther locule at stages 9 and 10. The expression of KCS20-GFP fusion protein was initially detected in *tapetum* at stage 9 and subsequently accumulated in anther locule and pollen at stages 10 and 11. GFP signal in *tapetum* was shown in an enlarged view. No GFP signals of KCS7-GFP, KCS15-GFP, and KCS21-GFP were identified in *ms1* background. T, *tapetum*. Bars = 20 μm. **(B)** The expression of the KCS10-GFP fusion protein was detected in the epidermis and endothecium at stages 10 and 11, while the GFP signal of KCS5 was detected in the epidermis at anther stages 10–12. Bars = 10 μm.

*Arabidopsis* genome contains 21 *KCS* genes. Of them, *KCS1/5/6/7/9/10/11/13/15/20/21* were reported to express in flowers (Joubès et al., 2008). We performed RT-PCR analysis and confirmed their expression in flowers (Supplementary Figure 1). Among them, *KCS5*, *KCS7*, and *KCS15* were specifically expressed in flowers, while *KCS6*, *KCS9*, *KCS10*, *KCS13*, *KCS20*, and *KCS21* were relatively highly expressed in flowers (Supplementary Figure 1). To further understand the expression patterns of these KCS proteins in the developing anthers, the *KCS* genomic sequences were fused to the *GFP* gene to make the reporter constructs (*pKCS:KCS-GFP*) to transform WT *Arabidopsis* plants. Transgenic lines expressing *pKCS:KCS-GFP* in anthers were identified (Figure 1A). The GFP signals of KCS7-GFP, KCS15-GFP, and KCS21-GFP proteins displayed similar expression patterns (Figure 1A). Their GFP fluorescence was detected in *tapetum* at anther stages 8–10 (Figure 1A) and in anther locule at anther stage 10 when *tapetum* PCD occurs. The expression of these *KCS*s is quite similar to that of tapetal sporopollenin synthesis genes (Wang et al., 2018). KCS20-GFP signal was also observed in *tapetum* at anther stage 9 and distributed within the anther locule following *tapetum* PCD. KCS5-GFP signal was localized in the epidermis at anther stages 10–12, while KCS10-GFP signal was preferentially detected in both epidermis and endothecium at anther stages 10 and 11 (Figure 1B). These demonstrate the expression of four *KCS* members, namely, *KCS7*, *KCS15*, *KCS20*, and *KS21*, in the *tapetum*.

### *KCS7*, *KCS15*, and *KCS21* Act Downstream of *MS1*

**FIGURE 2:**
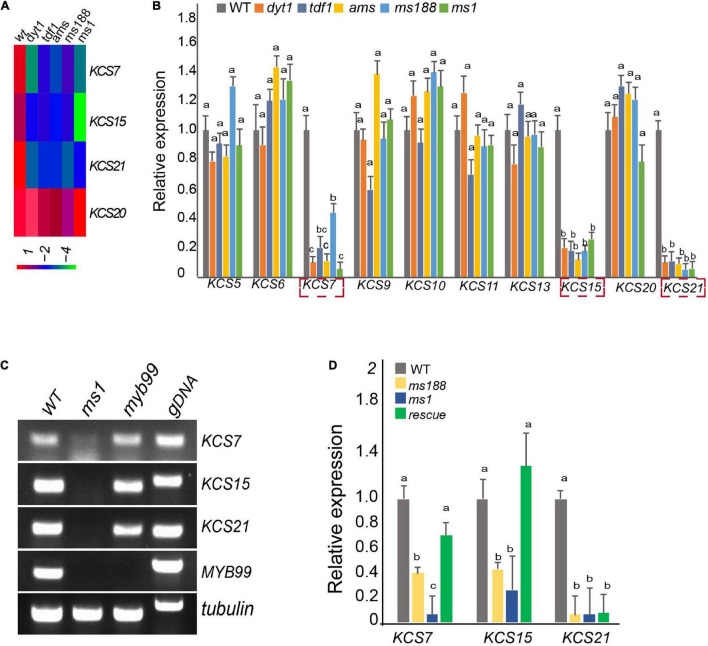
Male sterility 1 (MS1) mediates the expression of *KCS7*, *KCS15*, and *KCS21*. **(A)** Heatmap showing that the expression of *KCS7*, *KCS15*, and *KCS21* genes were downregulated in *dyt1*, *tdf1*, *ams*, *ms188*, and *ms1* mutants, while the expression of *KCS20* was not affected in these mutants. **(B)** qRT-PCR analysis of *KCS* genes in the inflorescence of WT, *dyt1*, *tdf1*, *ams*, *ms188*, and *ms1* mutants. Data are presented as mean and SD (*n* = 3). Letters above the bars indicate a significant difference between genotypes (one-way ANOVA test, *p* < 0.01). **(C)** RT-PCR analysis of *KCS7*, *KCS15*, and *KCS21* genes in *ms1* and *myb99* mutants. **(D)** Expression of *KCS7*, *KCS15*, and *KCS21* genes was analyzed in *pMS188:MS1* transgenic plants in the *ms188* homozygous background (rescue). Error bars show the SD (*n* = 3). Letters above the bars indicate a significant difference between genotypes (one-way ANOVA test, *p* < 0.01).

Dysfunctional tapetum 1, TDF1, aborted microspore (AMS), MS188, and MS1 are essential *tapetum* regulators that form a genetic pathway (Wilson et al., 2001; Sorensen et al., 2003; Zhang et al., 2006, 2007; Zhu et al., 2008, 2011). Based on the previous microarray data, *KCS7*, *KCS15*, and *KS21* act downstream of these regulators (Figure 2A; Zhu et al., 2011). We collected the inflorescences of *dyt1*, *tdf1*, *ams*, *ms188*, and *ms1* for quantitative real-time PCR (qPCR) analysis. The qPCR results showed that the expressions of *KCS7*, *KCS15*, and *KCS21* were downregulated, while *KCS20* was not affected in these mutants (Figure 2B).

Male sterility 1 is the key regulator for late *tapetum* development in the genetic pathway (Zhu et al., 2011). The downregulation of *KCS7*, *KCS15*, and *KCS21* in *ms1* mutant suggested that these three *KCS* genes act downstream of MS1. To further analyze the molecular regulation between MS1 and *KCS7*, *KCS15*, and *KCS21*, we introduced the *pKCS:KCS-GFP* construct into the *ms1* plants and analyzed the distribution of KCS-GFP signals (Figure 1A). The GFP signals of KCS7-GFP, KCS15-GFP, and KCS21-GFP could not be detected in *ms1* mutant while they were clearly observed in the tapetal cells of WT (Figure 1A). These results are consistent with the microarray and qPCR results (Figures 2A,B). *MYB99* is a direct target of MS1 and its mutant displays a slight defect in the pollen development (Alves-Ferreira et al., 2007). The expressions of *KCS7*, *KCS15*, and *KCS21* were not changed in the *myb99* mutant, indicating that these three *KCS* genes are not regulated by MYB99 (Figure 2C). MS188 is an upstream regulator of MS1 (Zhu et al., 2011). Our recent study showed that many anther *PCPs* are downregulated in both *ms188* and *ms1*, and the expression of *MS1* driven by the *MS188* promoter in *ms188* mutant (*ms188/pMS188:MS1*) restores their expression (Lu et al., 2020). We performed qRT-PCR in the same transgenic line (*ms188/pMS188:MS1*). The result showed that the expressions of *KCS7* and *KCS15* were fully restored in this transgenic plant (Figure 2D). However, the expression of *KCS21* did not show any recovery compared with that in the *ms1* mutant (Figure 2D). These results show that *KCS7*, *KCS15*, and *KCS21* act downstream of MS1 while the mechanism of their expression regulation may be varied.

### The Pollen Coat Is Reduced in *kcs7/15/21* Triple Mutant

**FIGURE 3:**
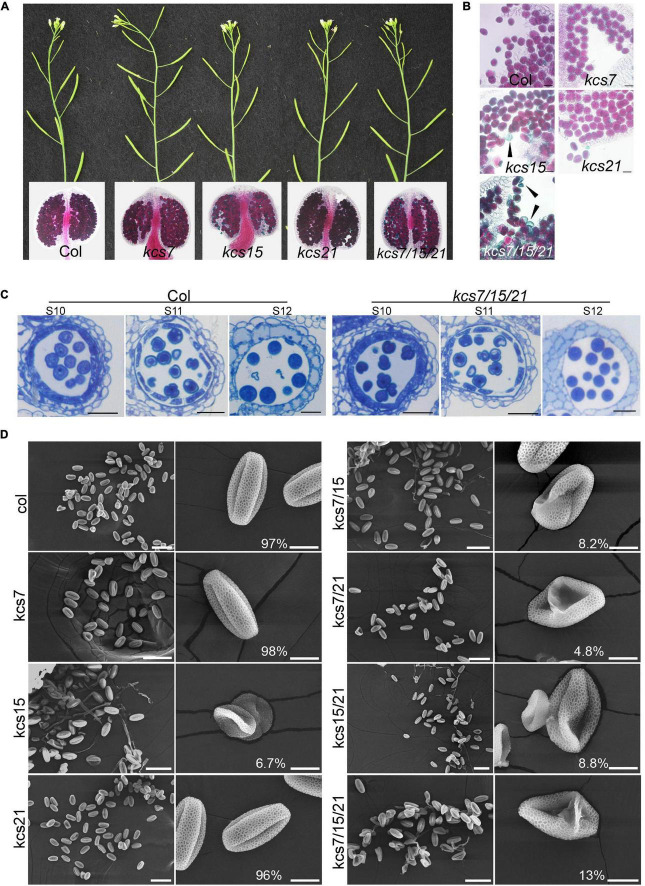
Phenotypes of single, double, and triple mutants of *kcs7*, *kcs15*, and *kcs21*. **(A,B)** Plants, anthers, and pollen grains of *kcs7*, *kcs15*, and *kcs21* single mutants and *kcs7*/*15*/*21* triple mutant. Black arrows indicate aborted pollen grains in *kcs15* and *kcs7-2*/*15*/*21-2* triple mutants. Bars = 20 μm. **(C)** Cell biological analyses of WT and *kcs7-2*/*15*/*21-2* triple mutant anthers using semi-thin transverse sections. Bars = 20 μm. **(D)** Pollen grains from the single, double, and triple mutants of the *KCS7*, *KCS15*, and *KCS21* genes were visualized under the scanning electron microscope (SEM). The representative phenotype of *kcs7-2*, *kcs15*, *kcs21-2*, *kcs7-2*/*15*, *kcs7-2*/*21-2*, *kcs15*/*21-2*, and *kcs7-2*/*15*/*21-2* was shown. The statistical number of the representative pollen grains was shown. The scale bar for huge pollen is 50 μm, and for single pollen it is 10 μm.

To investigate the functions of the *KCS7*, *KCS15*, and *KCS21* in the anther development, the mutants were obtained from TAIR resource^3^ with T-DNA being inserted in the exon of *KCS7*, *KCS15*, and *KCS21*, respectively (Supplementary Figure 2). No obvious vegetative or reproductive morphological abnormalities were observed in any of the single (*kcs7-1*, *kcs7-2*, *kcs15*, *kcs21-1*, and *kcs21-2*), double (*kcs7-1/15*, *kcs7-2/15*, *kcs7-1/21-1*, *kcs7-2/21-2*, *kcs15/21-1*, and *kcs15/21-2*), and triple mutants of *KCS7*, *KCS15*, and *KCS21* (*kcs7-1/15/21-1* and *kcs7-2/15/21-2*). Aborted pollen grains were occasionally observed in *kcs15* single mutant (70 out of 1,030), *kcs7-1/15* (42 out of 510), *kcs7-1/21-1* (36 out of 735), and *kcs15/21-1* (42 out of 473) double mutants (Figure 3 and Supplementary Figure 3). However, the statistical analyses showed that the percentage of aborted pollen grains was increased to 13% in *kcs7-1/15/21-1* (81 out of 630) and *kcs7-2/15/21-2* triple mutant (110 out of 853, Figure 3 and Supplementary Figure 3), indicating that *KCS7*, *KCS15*, and *KCS21* play redundant roles in the pollen development.

**FIGURE 4:**
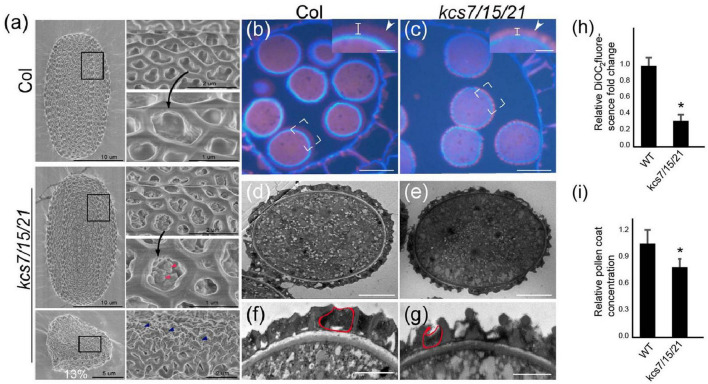
Pollen coat was affected in *kcs7*/*15*/*21* triple mutant. **(a)** SEM analysis of exine and pollen coat morphology in WT and *kcs7-2*/*15*/*21-2* triple mutant plants. Red arrows and blue arrows indicate irregular pollen coat and particles, respectively. **(b,c)** Lipid staining of semi-thin sections of WT and *kcs7-2*/*15*/*21-2* triple mutant plants at anther stage 12. The red fluorescence indicates the lipid staining on mature pollen grains (white arrow). Statistical analysis of pollen wall fluorescence of WT and *kcs7-2*/*15*/*21-2* triple mutant from 50 cross sections. Bars = 10 μm. **(d–g)** Analysis of pollen wall structure by transmission electron microscopy (TEM). The red circles in panels **(f,g)** indicate the outline of the pollen coat. Statistical analysis of the pollen coat of WT and *kcs7-2*/*15*/*21-2* triple mutant from 20 TEM sections. The ultrastructure of the pollen coat of *kcs7-2*/*15*/*21-2* triple mutant showed decreased and disordered compared with the wild type. **(h)** DiOC_2_ fluorescent intensity was calculated using Quantity One software (Bio-Rad, United States). Fold change indicates relative DiOC_2_ fluorescence (DiOC_2_ fluorescence intensity of WT was 1). Asterisk indicates a significant difference compared with the WT (Student’s *t*-test; *p < 0.01). **(i)** The relative pollen coat concentration was calculated using Quantity One software (Bio-Rad, United States). Fold change indicates relative pollen coat concentration (the pollen coat concentration of WT is 1). Asterisk indicates a significant difference compared with the WT (Student’s *t*-test; *p < 0.05). Bars = 5 μm in panels **(d,e)** and bars = 1 μm in panels **(f,g)**.

Given that both *kcs7-1/15/21-1* and *kcs7-2/15/21-2* triple mutants displayed a similar defect in pollen grains, *kcs7-2/15/21-2* was used for further analysis. Lipids are deposited in the pollen coat during pollen development, and KCS proteins are responsible for lipid synthesis. To analyze whether mutation in *KCS7*, *KCS15*, and *KCS21* affects the pollen coat lipids, the SEM and TEM observations were used. The WT pollen grains are uniformly spheroid (13.6 μm in diameter) with a well-organized exine and a smooth pollen coat. However, in the WT pollen grains of the triple mutant, the well-smoothed pollen coat is disturbed, with many particles deposited in the cavity of pollen exine (Figure 4). In addition, approximately 13% (98 out of 760) of the triple mutant pollen grains are collapsed with a shrunken morphology (6.3 μm in diameter) and displayed disorganized exine covered with additional unknown materials (Figure 4). The DiOC_2_ was used to stain the fatty acid content of the pollen wall, which exhibits a red fluorescence (Gu et al., 2014). The WT mature pollen grains are deeply stained by DiOC_2_ with strong red fluorescence in exine and pollen coat (Figures 4b,c,h), whereas *kcs7/kcs15/kcs21* mature pollen shows weaker pink signals on pollen walls. In addition, compared with the WT pollen, the fluorescence signal is much patchy and the relative fluorescence fold change is low in *kcs7/kcs15/kcs21* pollen walls (Figures 4b,c,h). In contrast to the regular arrangement of pollen coat in the WT exine (Figures 4d,f,i), phenotypic and statistical analyses showed that the *kcs7/kcs15/kcs21* mutant displayed a decreased pollen coat with a disordered arrangement (Figures 4e,g,i). Therefore, these results indicate that lipid content is reduced in the pollen coat of *kcs7/kcs15/kcs21*.

### Pollen Hydration Is Delayed in *kcs7/15/21* Triple Mutant

**FIGURE 5:**
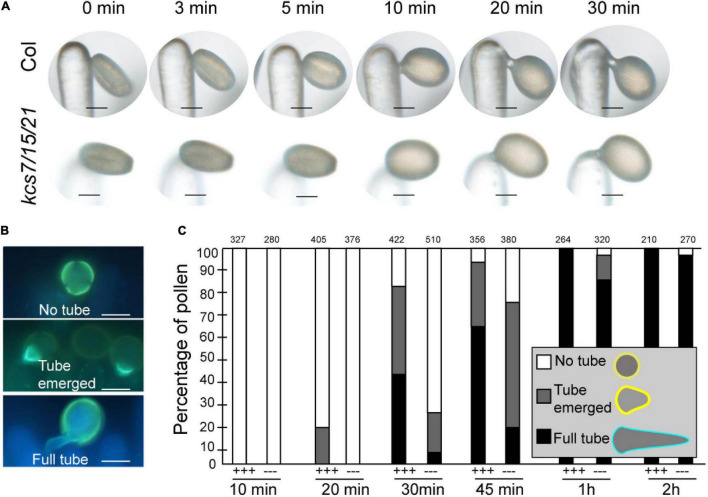
*In vivo* pollen hydration and pollen tube growth analysis in *kcs7*/*15*/*21* triple mutant. **(A)** Diagram of pollen hydration in WT and *kcs7-2*/*15*/*21-2* triple mutant. **(B)** Diagram of pollen germination on stigma. **(C)** *In vivo* pollen tube emergence after manual pollination at 10, 20, 30, 45 min, 1 and 2 h post-pollination. Pollen tubes were formed in WT pollen after manual pollination at 20 min, while pollen tubes were identified in *kcs7-2*/*15*/*21-2* triple mutant at 30 min. Pollen grains were applied to stigmas of *ms188*, and the statistical information was shown. +++, WT pollen; −−−, *kcs7-2*/*15*/*21-2* mutant pollen. Bars = 10 μm.

Pollen coat is generally considered to involve in pollen-stigma interaction. A pollen hydration assay was carried out to analyze if the reduced pollen coat in *kcs7/kcs15/kcs21* affects the pollen-stigma interaction. *Ms188* is a male sterile line without any pollen inside anther while its stigma is not affected (Zhang et al., 2007). The pollen hydration assay was carried out by pollinating stigmas of *ms188* with pollen grains from WT and the *kcs7/15/21* triple mutant (Figure 5). After landing on stigma, the WT pollen began to absorb water and became spherical (pollen hydration) in about 5 min (Figure 5A). However, the hydration of the *kcs7/15/21* triple mutant pollen occurred in about 10 min after pollination. Accordingly, pollen germination of the triple mutant is also delayed (Figure 5A). To determine whether the hydration delay is derived from a defect in water absorption, an *in vitro* hydration of WT and *kcs7/15/21* triple mutant pollen in PEG 3350 series was carried out (Supplementary Figure 4). The *in vitro* hydration was not significantly different between WT and *kcs7/15/21* triple mutant pollen in PEG 3350 series, indicating that the absence of KCS7, KCS15, and KCS21 proteins did not impair the ability of pollen to absorb water. To test whether the subsequent steps of pollination were also affected in *kcs7/15/21* triple mutant, we monitored pollen tube initiation and growth as previously described (Mayfield and Preuss, 2000; Supplementary Figure 5 and Figure 5B). About 20% of WT pollen grains showed a pollen-tube emergence within 20 min of pollination, while *kcs7/15/21* triple mutant pollen-tube emergence was postponed to 30 min (Figure 5C and Supplementary Figure 5). After 2 h, WT pollen produced significantly longer tubes (470 μm) and penetrated into the style. However, pollen tubes of *kcs7/15/21* triple mutant were much shorter (266 μm) (Supplementary Figure 5). Therefore, the pollen tube elongation is postponed in *kcs7/15/21* triple mutant (Figures 5B,C, and Supplementary Figure 5). Despite the observed defects in pollen hydration and pollen tube elongation in *kcs7/15/21* triple mutant, there was no significant difference in seed set in *kcs7/15/21* triple mutant compared with WT plants.

### The Expression of KCS6 in Endothecium Persists Much Later Than KCS7/15/21 in *Tapetum* During Anther Development

**FIGURE 6:**
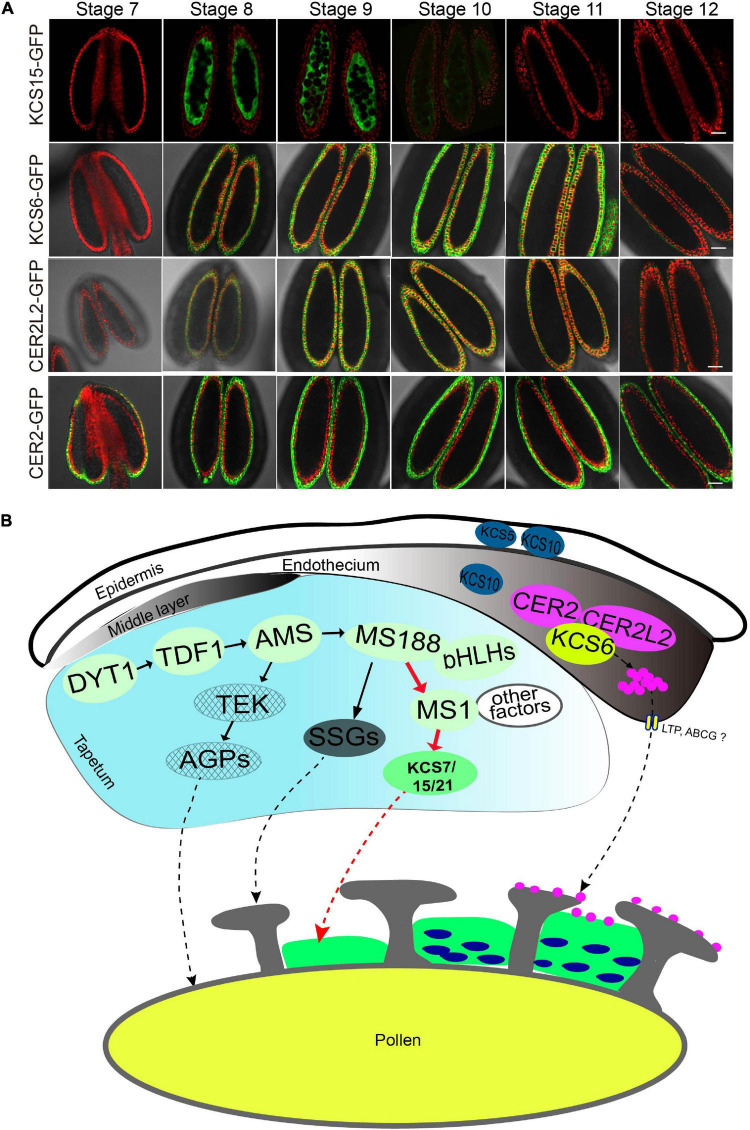
A proposal model for anther-derived lipids in pollen wall development. **(A)** Expression pattern of KCS6, KCS15, CER2, and CER2L2 proteins during anther development. The expression of KCS15-GFP was detected in *tapetum*, while the expression of KCS6-GFP, CER2-GFP, and CER2L2-GFP fusion proteins were detected in endothecium. Bars = 20 μm. **(B)** A proposed model for *tapetum* and endothecium in the biosynthesis of lipids for pollen wall development. In *tapetum*, MS1 might regulate the expression of *KCS7*, *KCS15*, and *KSC21*, and the KCS-mediated lipids were deposited in pollen coat. In endothecium, CER2 and CER2L2 interact with KCS6 to modulate the biosynthesis of pollen coat VLCFAs for pollen hydration.

Our recent study showed that KCS6 interacts with CER2/CER2L2 for the synthesis of pollen coat VLCFAs which is essential for pollen hydration (Zhan et al., 2018). Therefore, both KCS6 in endothecium and KCS7/15/21 in *tapetum* might contribute to the synthesis of pollen coat lipids. Currently, no efficient techniques are available to distinguish the difference between lipids synthesized by the *tapetum* and endothecium, respectively. We compared the expression of KCS and CER proteins during the anther development to understand the accumulation of the lipids in the pollen coat. In a previous study, we obtained transgenic lines of *pKCS6:KCS6-GFP*, *pCER2:CER2-GFP*, and *pCER2L2:CER2L2-GFP* (Zhan et al., 2018). All of the GFP transgenic plants were grown in the same pot and were cultured in the same condition. KCS6, CER2, and CER2L2 displayed a longer period expression in endothecium at anther stages 8–12 (Figure 6A). KCS7, KCS15, and KCS21 were expressed in *tapetum* (stages 8–10) (Figures 1, 6). They displayed a shorter period expression than KCS6, CER2, and CER2L2 expressed in endothecium (Figure 6A). These results suggested that the *tapetum*-derived lipids are synthesized and deposited in the pollen coat much earlier than endothecium-derived lipids. MS1 protein accumulates in tapetal cells at anther stages 7 and 8 (Yang et al., 2007), overlapping with that of KCS7, KCS15, and KCS21 (Figure 6A), further indicating that the expression of *KCS7*, *KCS15*, and *KCS21* is dependent on MS1 (Figure 2).

### 3-Ketoacyl-Coenzyme A Synthases Proteins Are Evolutionarily Widespread

**FIGURE 7:**
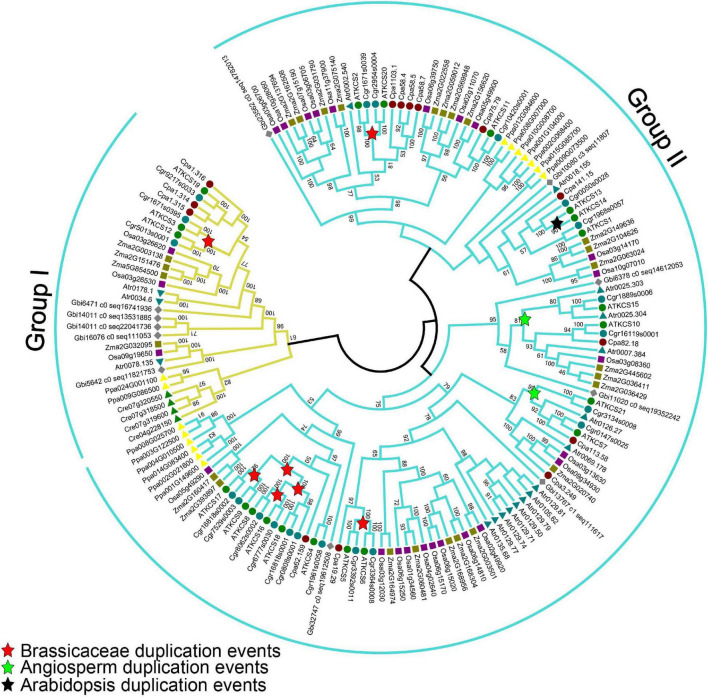
Maximum-likelihood phylogenetic analysis of KCS proteins from various plant lineages. The analysis was generated with 1,000 bootstrap replications using the full-length amino acids. Bootstrap values are indicated at nodes within the tree. The red, green, and blue stars represent duplication events in Brassicaceae, angiosperm, and *Arabidopsis*, respectively. AT, *Arabidopsis thaliana*; Atr, *Amborella trichopoda*; Cre, *Chlamydomonas reinhardtii*; Cgr, *Capsella grandiflora*; Cpa, *Carica papaya*; Gbi, *Ginkgo biloba*; Osa, *Oryza sativa*; Ppa, *Physcomitrella patens*; Zma, *Zea mays*.

To determine the evolutionary relationships among the KCS proteins, an unrooted ML phylogenetic tree was constructed across the known plant lineages (Figure 7). The number of copies of *KCS* genes varied considerably among plants, ranging from 4 in the green algae *C. reinhardtii* to 15 in *P. patens* (Bryophyta), 21 in *A. thaliana* (eudicot), 25 in *O. sativa*, and 26 in *Z. mays* (monocot). More *KCS* gene copies were identified in land plants than in algae, indicating that duplication of *KCS* genes likely occurred after land plants split from green algae. Based on the phylogenetic analyses, the KCS proteins can be divided into two subgroups, with group I containing AtKCS3, AtKCS12, AtKCS19, and the remaining proteins belonging to group II. In addition to *Arabidopsis* KCS proteins, different duplication events were identified, where 12 KCS (KCS 2, 3, 4, 5, 6, 8, 9, 12, 16, 17, 18, and 20) proteins were produced by independent duplications in Brassicaceae, four KCS (i.e., KCS7, KCS10, KCS15, and KCS21) proteins were originated from the duplication events that occurred in the ancestor of angiosperm, and two KCS (i.e., KCS13, KCS14) proteins were originated from independent duplication in *Arabidopsis* (Figure 7). These duplication events may reflect functional specialization of *Arabidopsis* KCS proteins. In addition, many monocot-specific duplication events were identified in rice and maize (Figure 7), indicating the functional specification of KCS proteins between dicots and monocots.

## Discussion

### Pollen Coat Lipids Derived From Both *Tapetum* and Endothecium

Pollen coat lipids are a major part of the pollen coat, which constitutes the outer layer of pollen. Previous investigation suggests that some pollen coat lipids are derived from endothecium (Zhan et al., 2018). Generally, it was considered that pollen coat lipids are mainly derived from *tapetum* (Hernández-Pinzón et al., 1999). In this study, the reduced pollen coat lipids in the triple mutant of *tapetum* expressed genes (*KCS7*, *KCS15*, and *KCS21*) suggest that these *KCS*s play a role in *tapetum* to provide pollen coat lipids (Figures 4, 6). KCS7, KCS15, and KCS21 were expressed in *tapetum* from stages 8 to 10 and were released to anther locule following *tapetum* PCD (Figure 1). It is likely that lipids begin to deposit outside the developing microspores before *tapetum* PCD. With *tapetum* PCD, all *tapetum* compounds, including lipids, deposit in the pollen coat. In *kcs6* mutant, the amount of part VLCFAs (C_29_ and C_30_ lipids) was decreased in pollen coat (Fiebig et al., 2000), indicating that KCS6-mediated VLCFAs are deposited in pollen coat. The expression of KCS6, CER2, and CER2L2 in endothecium is much later than the expression of KCS7, KCS15, and KCS21 in *tapetum* (Figure 6A). These further support the previous possibility that lipids from endothecium deposit outside the lipids from *tapetum* (Zhan et al., 2018). KCSs catalyze the synthesis of C20 to C28 lipids, while CER2 and CER2L2 are required for the production of C28 to C34 lipids (Fiebig et al., 2000; Haslam et al., 2015). This indicates that the inner pollen coat lipid from *tapetum* is likely medium- and long-chain fatty acids (<28 carbon atoms), while the outer pollen coat lipid from endothecium is VLCFAs (>30 carbon atoms).

### Pollen Coat Lipids Function in Pollen-Stigma Interaction

The pollen-stigma interaction includes pollen adhesion, hydration, and pollen tube growth. The pollen coat lipids may display a semisolid state and waterproof the pollen grain from its dispersal to its capture on a compatible stigma (Wheeler et al., 2001). Following the pollen-stigma interactions, a “footlike” structure is formed to enhance pollen-stigma adhesion (Elleman and Dickinson, 1990). During the “foot” formation, the lipids may create a capillary system to facilitate water transfer from the stigma cell to desiccated pollen, and pollen hydration occurs. However, it is difficult to separate clearly the processes of pollen adhesion and hydration in most reports. The VLCFAs from endothecium is predicted to locate the outer pollen coat (Zhan et al., 2018). In the mutant of *kcs6* and *cer2cer2l2*, the pollen could not hydrate on stigma, resulting in male sterility of the plants (Fiebig et al., 2000; Haslam et al., 2015; Zhan et al., 2018). This hydration defects might result from adhesion defects. The delayed hydration in the triple mutant of *kcs7/15/21* suggests that the *tapetum*-derived lipids might assist in establishing a water gradient between the pollen and stigma after pollen adhesion (Figure 5), and its mutation only postpone the pollen hydration (Figure 5B). We propose that the endothecium-derived lipid might function early in pollen adhesion, while *tapetum*-derived lipid might function later in pollen hydration. This hypothesis needs to be studied further. Hydration defects have also been reported in mutants of PCPs, such as EXL4, GRP17, and PCP-B (Wolters-Arts et al., 1998; Updegraff et al., 2009; Wang et al., 2017), indicating that lipids and proteins of pollen coat may work cooperatively to facilitate pollen hydration.

### The *Tapetum* Regulatory Cascade for Pollen Coat Lipid Formation

*Tapetum* provides nutrition for microspore development, secrets hydrolases for tetrad wall dissolution, and supplies materials for pollen wall and pollen coat formation during anther development. In *Arabidopsis*, the genetic regulatory pathway DYT1-TDF1-AMS-MS188-MS1 is important for *tapetum* development and function (Zhu et al., 2011; Gu et al., 2014; Lou et al., 2014, 2018). In this pathway, AMS directly regulates ABCG26 for sporopollenin transportation (Xu et al., 2010) and MGT5 to provide the Mg2^+^ for microspores development (Xu et al., 2015). MS188 directly regulates CYP703A2 and other sporopollenin biosynthesis genes for sporopollenin synthesis and sexine formation (Xiong et al., 2016; Wang et al., 2018). Sexine is the outer pollen wall where pollen coat (including pollen coat lipids) is deposited. MS1 is a transcription factor for late *tapetum* development directly regulated by MS188. It regulates the expression of multiple PCPs (Lu et al., 2020). MS1 also affects the expression of *KCS7*, *KCS15*, and *KCS21* for pollen coat lipid synthesis (Figures 2, 6). This pollen coat lipid may provide a matrix for the assembly of pollen coat PCPs (Figure 6B). After *tapetum* PCD, the endothecium-derived lipids might be transported and deposited outside of the pollen coat (Figure 6B). Together, this study reveals that following outer pollen wall formation regulated by MS188, its directly regulating transcription factor MS1 modulates the downstream gene expression for pollen coat formation. This regulatory cascade is helpful to make sure that pollen wall and pollen coat are orderly synthesized during anther development.

## Data Availability Statement

The original contributions presented in the study are included in the article/Supplementary Material, further inquiries can be directed to the corresponding author/s.

## Author Contributions

Z-NY, Z-BZ, and H-DZ planned and designed the research and wrote the manuscript. Z-BZ and H-DZ were involved with all aspects of the research with S-XX. J-YL and N-YY contributing to expression analysis, SEM, and TEM work. H-YY assisted with critical assessment of the manuscript. All authors contributed to the article and approved the submitted version.

## Conflict of Interest

The authors declare that the research was conducted in the absence of any commercial or financial relationships that could be construed as a potential conflict of interest.

## Publisher’s Note

All claims expressed in this article are solely those of the authors and do not necessarily represent those of their affiliated organizations, or those of the publisher, the editors and the reviewers. Any product that may be evaluated in this article, or claim that may be made by its manufacturer, is not guaranteed or endorsed by the publisher.

## Footnotes

1
https://phytozome.jgi.doe.gov/pz/portal.html

2
http://smart.embl-heidelberg.de/

3
www.arabidopsis.org

## Supplementary Material

Supplementary Figure 1 Reverse transcription-PCR (RT-PCR) expression analysis of 11 *KCS* genes in root, stem, leaf, and flowers.

Supplementary Figure 2 The T-DNA locations of detected genes.

Supplementary Figure 3 The statistical rate of the aborted pollen grains in different mutants. Error bars show the SD (*n* = 3). Asterisks indicate significantly different means (*p* < 0.05) using one-way ANOVA test.

Supplementary Figure 4 Phenotype **(A)** and statistic width **(B)** of *kcs7-2*/*15*/*21-2* triple mutant and wild-type pollen when placed in a PEG 3350 series. Each point represents the average width of 100 pollen grains.

Supplementary Figure 5 *kcs7-2*/*15*/*21-2* triple mutant and wild-type *in vivo* pollen tube growth. **(A)** The emergence of pollen tube with aniline blue staining. Scale bar represents 10 μm in figures of 10, 20, 30, and 45 min, 80 μm in figure of 1 h, 100 μm in figure of 2 h. **(B)** The length of pollen tubes at different hours.

Supplementary Table 1 Primers used in this study.
