# Supplementary figures and images for: Tapetal 3-Ketoacyl-Coenzyme A Synthases Are Involved in Pollen Coat Lipid Accumulation for Pollen-Stigma Interaction in Arabidopsis
Source: Front Plant Sci. 2021 Nov 23;12:770311. doi: 10.3389/fpls.2021.770311 (PMC8650583; doi:10.3389/fpls.2021.770311)

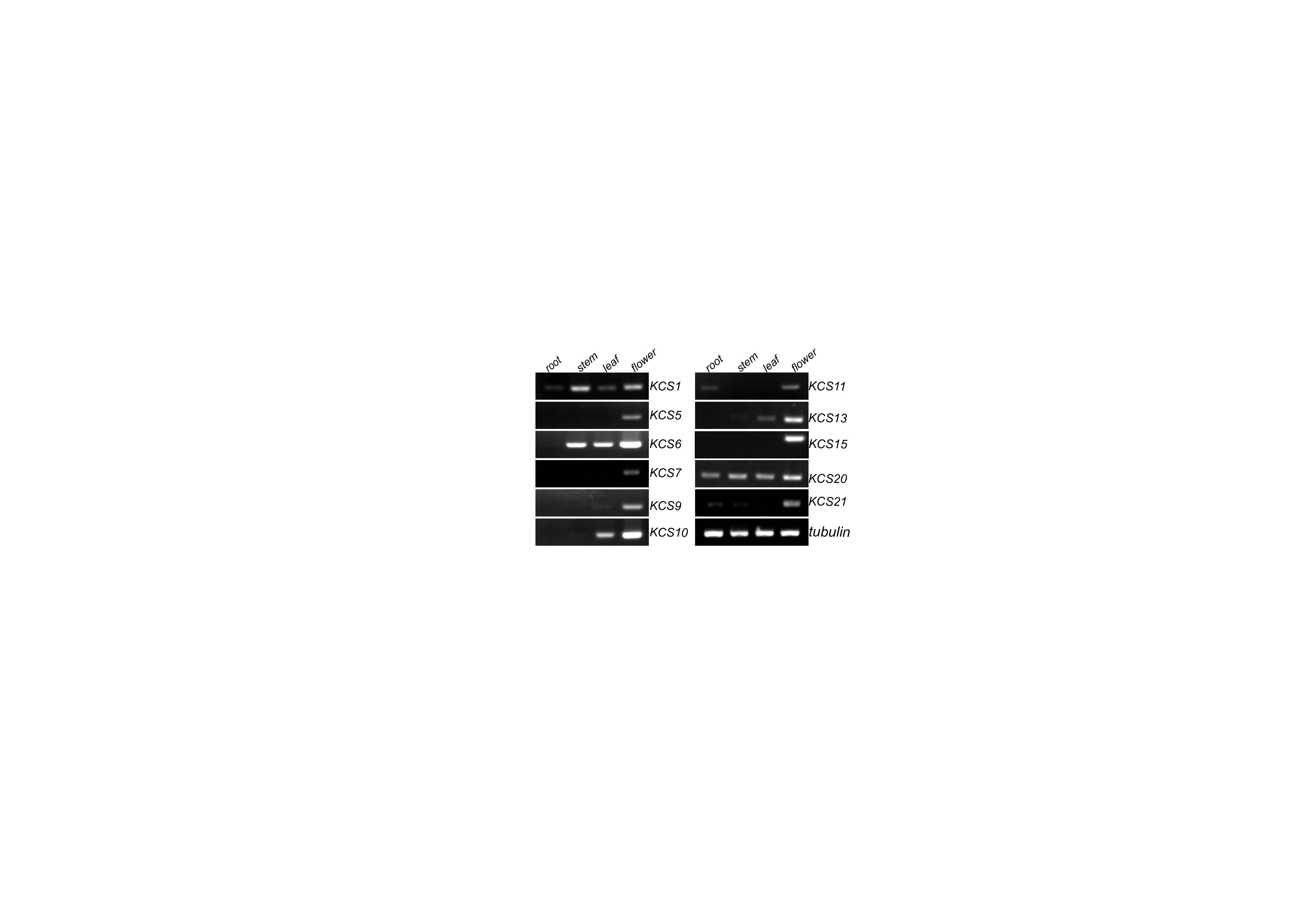

Supplement: Supplementary Figure 1 — Reverse transcription-PCR (RT-PCR) expression analysis of 11 KCS genes in root, stem, leaf, and flowers. [file Image_1.JPEG]

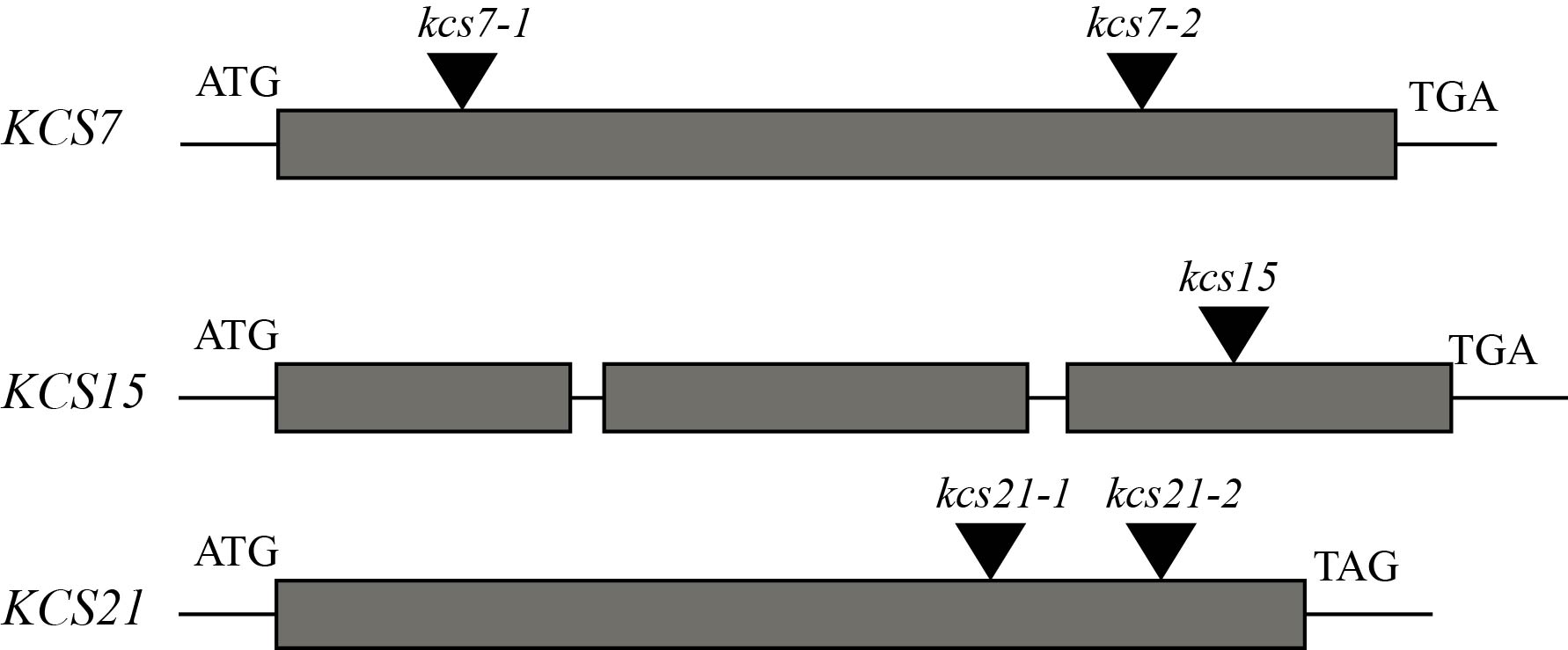

Supplement: Supplementary Figure 2 — The T-DNA locations of detected genes. [file Image_2.JPEG]

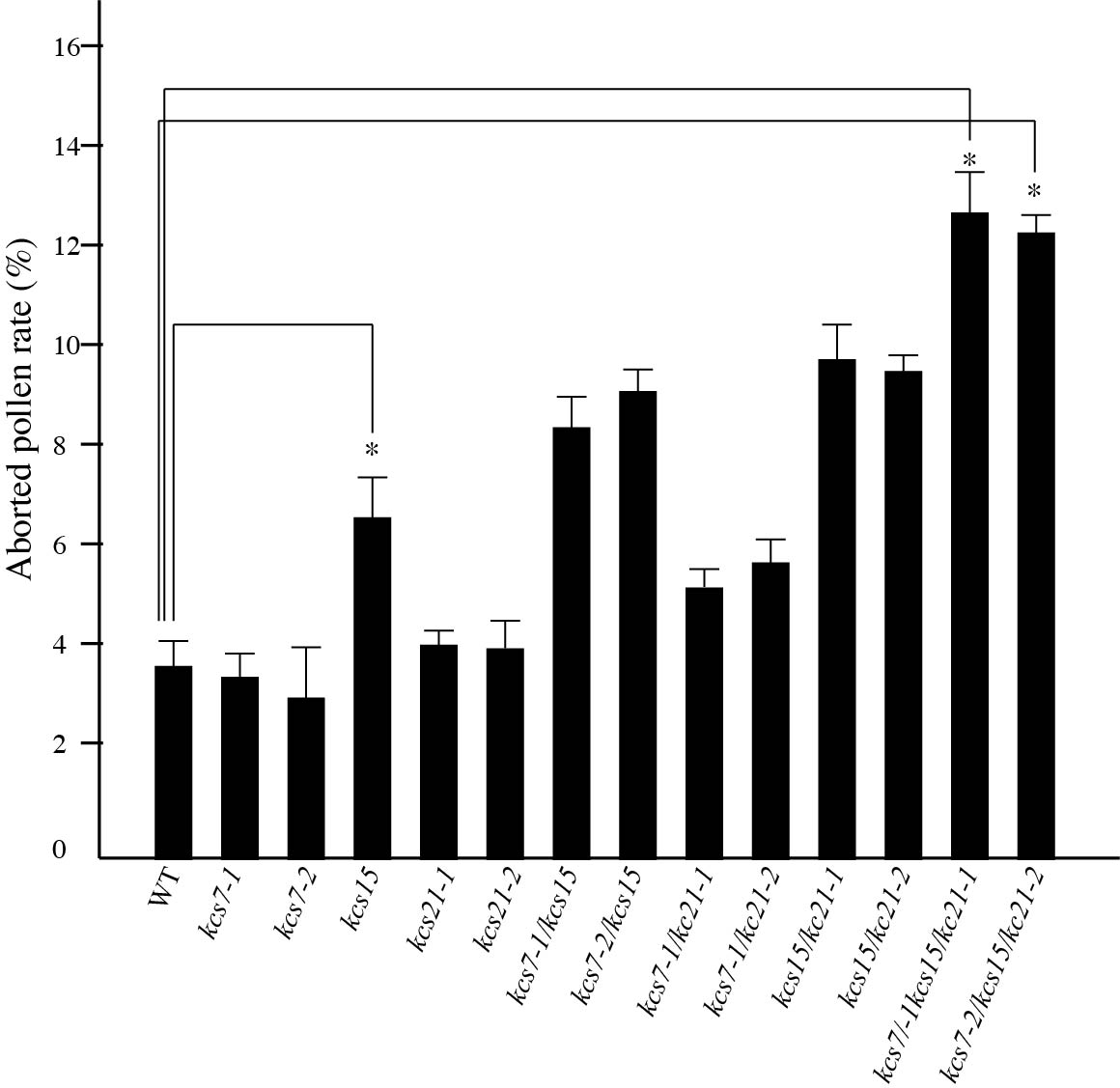

Supplement: Supplementary Figure 3 — The statistical rate of the aborted pollen grains in different mutants. Error bars show the SD (n = 3). Asterisks indicate significantly different means (p < 0.05) using one-way ANOVA test. [file Image_3.JPEG]

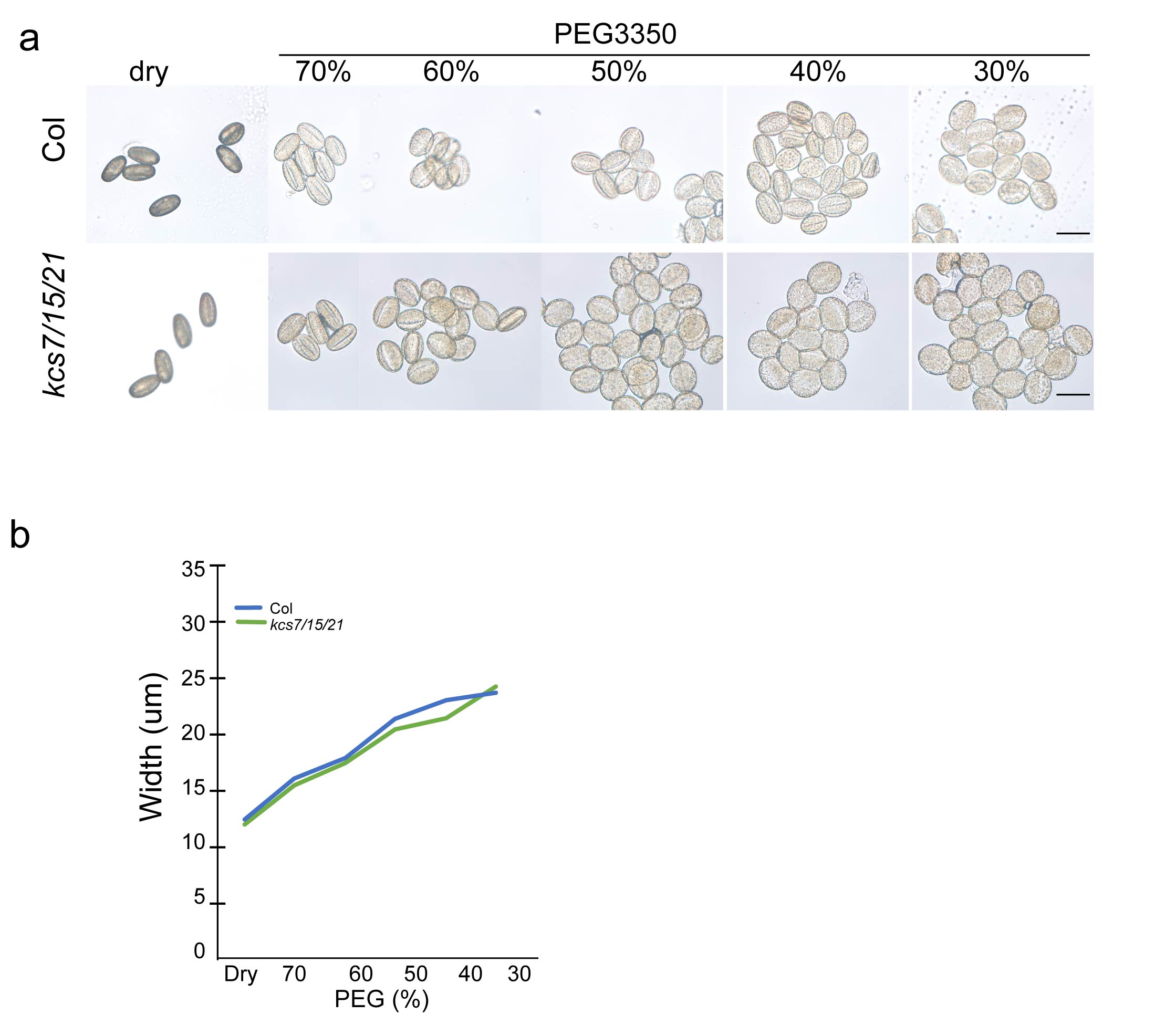

Supplement: Supplementary Figure 4 — Phenotype (A) and statistic width (B) of kcs7-2/15/21-2 triple mutant and wild-type pollen when placed in a PEG 3350 series. Each point represents the average width of 100 pollen grains. [file Image_4.JPEG]

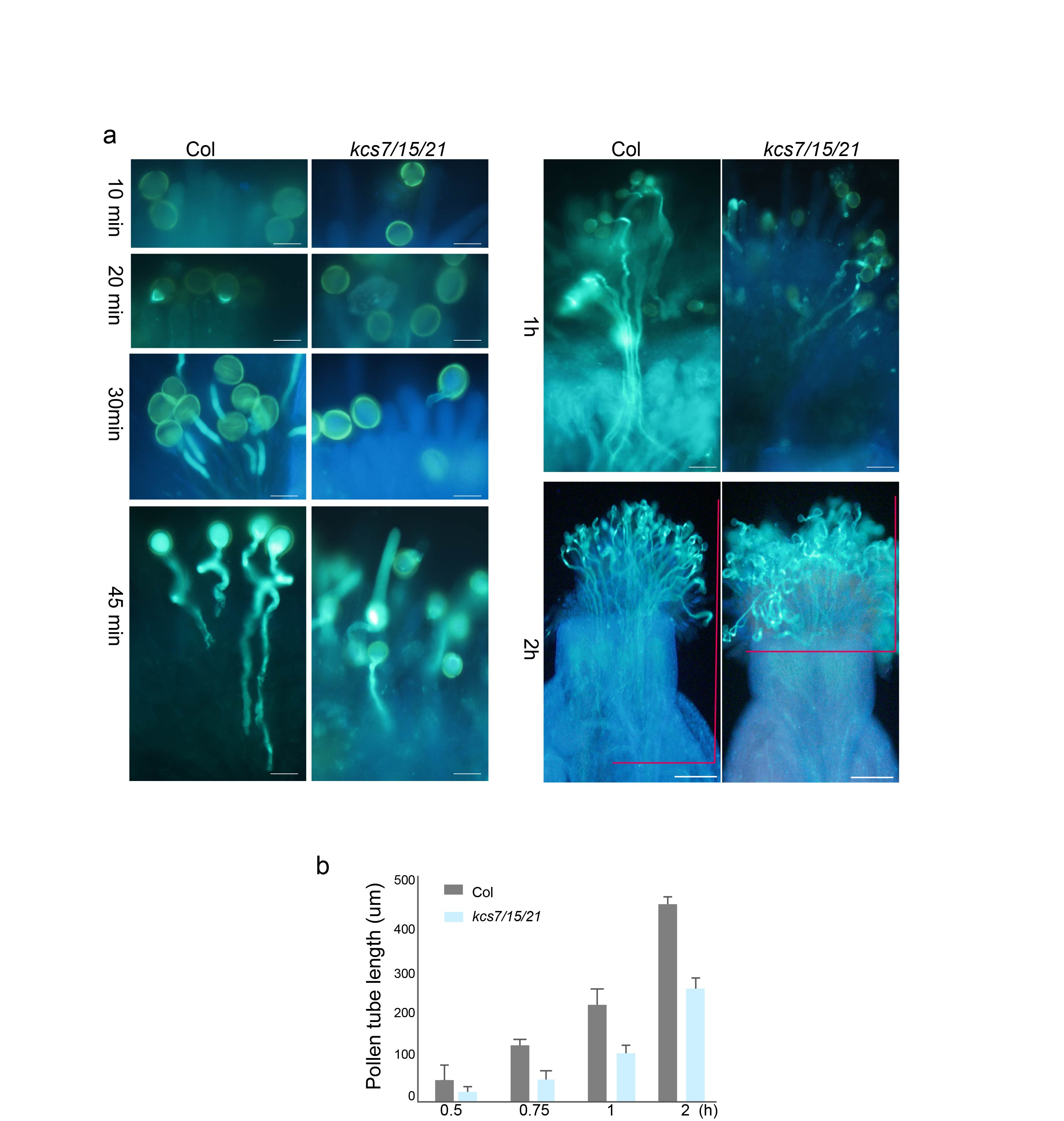

Supplement: Supplementary Figure 5 — kcs7-2/15/21-2 triple mutant and wild-type in vivo pollen tube growth. (A) The emergence of pollen tube with aniline blue staining. Scale bar represents 10 μm in figures of 10, 20, 30, and 45 min, 80 μm in figure of 1 h, 100 μm in figure of 2 h. (B) The length of pollen tubes at different hours. [file Image_5.JPEG]
